# Co-Immobilization of Enzymes and Magnetic Nanoparticles by Metal-Nucleotide Hydrogelnanofibers for Improving Stability and Recycling

**DOI:** 10.3390/molecules22010179

**Published:** 2017-01-23

**Authors:** Chunfang Li, Shuhui Jiang, Xinying Zhao, Hao Liang

**Affiliations:** 1 Department of Environment Protection and Detection, Beijing Industrial Technician College, Beijing 100023, China; bitclichunfang@163.com; 2 State Key Laboratory of Chemical Resource Engineering, Beijing University of Chemical Technology, Beijing 100029, China; 18810976705@163.com; 3 Beijing Centre for Physical and Chemical Analysis, Beijing 100089, China; Zhaoxinying_123@sina.com

**Keywords:** metal, nucleotide, magnetic iron oxide nanoparticles, *Candida rugose* lipase (CRL), immobilized enzyme

## Abstract

In this paper we report a facile method for preparing co-immobilized enzyme and magnetic nanoparticles (MNPs) using metal coordinated hydrogel nanofibers. *Candida rugosa* lipase (CRL) was selected as guest protein. For good aqueous dispersity, low price and other unique properties, citric acid-modified magnetic iron oxide nanoparticles (CA-Fe_3_O_4_ NPs) have been widely used for immobilizing enzymes. As a result, the relative activity of CA-Fe_3_O_4_@Zn/AMP nanofiber-immobilized CRL increased by 8-fold at pH 10.0 and nearly 1-fold in a 50 °C water bath after 30 min, compared to free CRL. Moreover, the immobilized CRL had excellent long-term storage stability (nearly 80% releative activity after storage for 13 days). This work indicated that metal-nucleotide nanofibers could efficiently co-immobilize enzymes and MNPs simultaneously, and improve the stability of biocatalysts.

## 1. Introduction 

Enzymes have been considered as potential substitutes for traditional chemical catalysts due to their environmental friendliness [1], low cost [2], high specificity [3], and mild reaction conditions [4]. As important biocatalysts, the use of enzymes is widespread in many applications, such as biosensors [5], pharmaceuticals, chemicals and foods [6,7]. However, some deficiencies, such as short lifetime and low operational stability have seriously limited their application [8,9,10,11]. Enzyme immobilization techniques play an important role in overcoming these obstacles [12]. What’s more, enzymes immobilized on solid supports show improved essential properties in actual application. Various materials have been used for immobilizing enzymes [13], such as carboxyl-functionalized graphene oxide [14], polyurethane foam support [15], SBA-15 [16], and chitosan [17]. Nowadays, the exploration of novel support materials and technologies are still attracting much attention.

Nanostructured carriers have high specific surface area [18], excellent dispersibility, and low mass transfer resistance, being considered as ideal materials for immobilizing enzymes [19,20,21]. To enhance enzymatic stability and activity, many nanomaterials have been used for immobilizing enzymes [22]. Compared with micro-scale carriers, nonporous nanoparticles are more flexible for reactors and rigid enough to withstand high pressure during their application, but they are quite fragile and can be frayed and hardened by dehydration or an intense crosslinking [23]. That’s why most researchers select porous nanomaterials as immobilized supports nowadays. Besides, the difficult recovery of nanostructure materials also limits their recycling, which significantly increases the cost. For solving this problem, magnetic nanosupports would be the best choice to improve the recovery of nanostructured immobilized enzymes by using magnets as the separation medium [24,25]. Thus, magnetic nanoparticles, such as iron oxide and ferric oxide, have been successfully introduced into immobilizing composites. In these studies, functionalized magnetic nanoparticles have been used for immobilization [26], including graphene-Fe_3_O_4_ [27], silica-Fe_3_O_4_ [28], and polymer grafted-Fe_3_O_4_ [29,30]. However, most of these methods are complicated to operate and easily lead to inactivation of the biocatalyst, so a proper immobilization support can improve enzyme operational stability (via multipoint or multisubunit attachment), activity, specificity, lifetimes, productivity, structural rigidity, and even provided reduction of inhibition [31,32,33,34,35].

Nucleotide-hybrid metal coordination polymers (NMCPs) have been widely used to prepare metal-organic frameworks [36], nanoparticles [37,38,39,40] and hydrogels [41,42]. More importantly, NMCPs have excellent adaptive self-assemble properties and can entrap various guest molecules, including proteins and nanoparticles. We recently demonstrated that Zn^2+^/AMP CPs could efficiently immobilize single enzymes or multi-enzymes with high stability [43]. Because of their nanofibrous properties, it’s very difficult to recover these immobilized enzymes by traditional methods. Thus, it would be very interesting to construct Zn^2+^/AMP nanofibers with magnetic response.

**Scheme 1:**
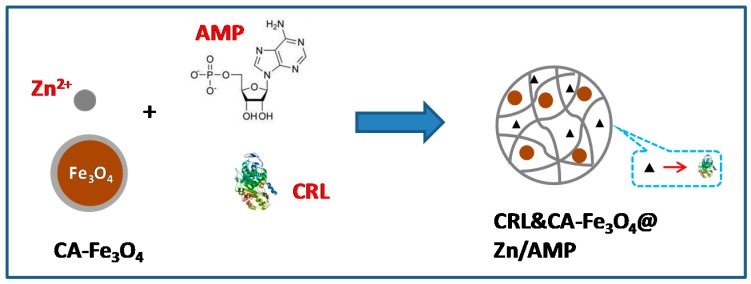
A scheme for preparing co-immobilized CRL and Fe_3_O_4_ NPs by the self-assembly of Zn^2+^ and AMP.

Lipases are prominent biocatalysts to catalyze reactions in diversified areas [44]. *Candida rugosa* lipase (CRL) is a versatile enzyme to convert various substrates into useful products [17]. Herein, we reported a convenient, efficient, and high capacity magnetic immobilization method for enzymes by the co-entrapment of Fe_3_O_4_ nanoparticles and CRL simultaneously within Zn/AMP nanofiber supports (Scheme 1). We characterized the as-prepared composites and successfully proved CRL and CA-Fe_3_O_4_@Zn/AMP NPs have high stability activity and good recovery.

## 2. Results and Discussion

**Figure 1:**
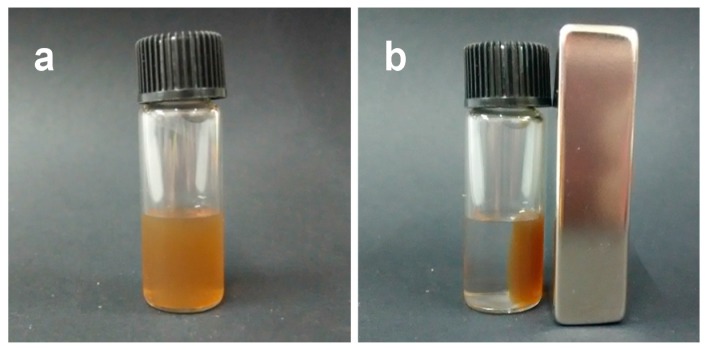
The magnetic separation behavior of CA-Fe_3_O_4_@Zn/AMP CPs. (**a**) Before; (**b**) After.

First, the entrapping properties of Zn/AMP nanofibers for Fe_3_O_4_ NPs were tested. Citric acid- modified Fe_3_O_4_ NPs (CA-Fe_3_O_4_ NPs) were synthesized by the reported method [45]. Briefly, AMP (25 mM) and CA-Fe_3_O_4_ NPs (5 mg/mL) were added into HEPES buffer (10 mM, pH 7.4). Then, ZnCl_2_ (50 mM) was mixed into the above mixture, and a brown precipitate appeared after several minutes. After 1 h, the magnetic Zn/AMP nanofibers were collected with an external magnet (Figure 1). The clear supernatant indicated that CA-Fe_3_O_4_ NPs were successfully entrapped into the Zn/AMP nanofibers.

**Figure 2:**
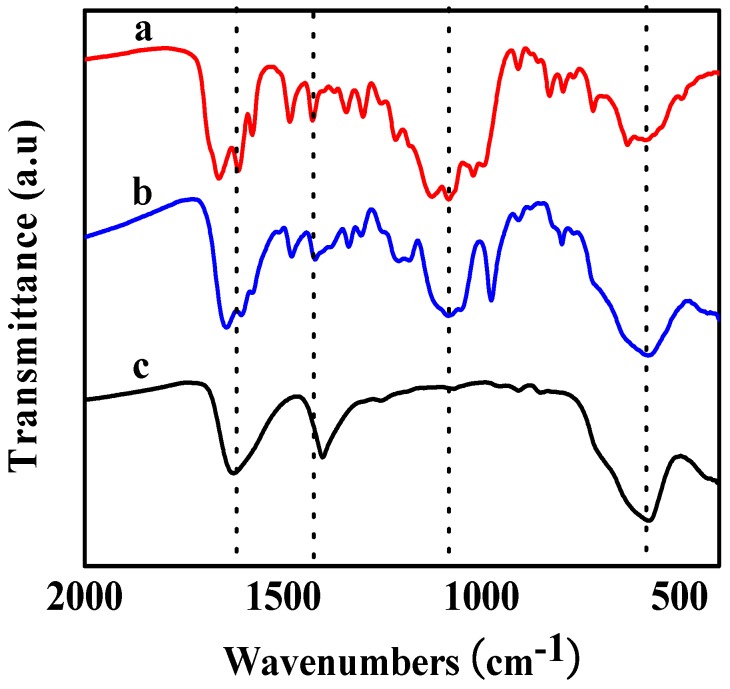
The FTIR spectra of CA-Fe_3_O_4_@Zn/AMP (curve **a**), CA-Fe_3_O_4_ & AMP (curve **b**) and CA-Fe_3_O_4_ NPs (curve **c**).

**Figure 3:**
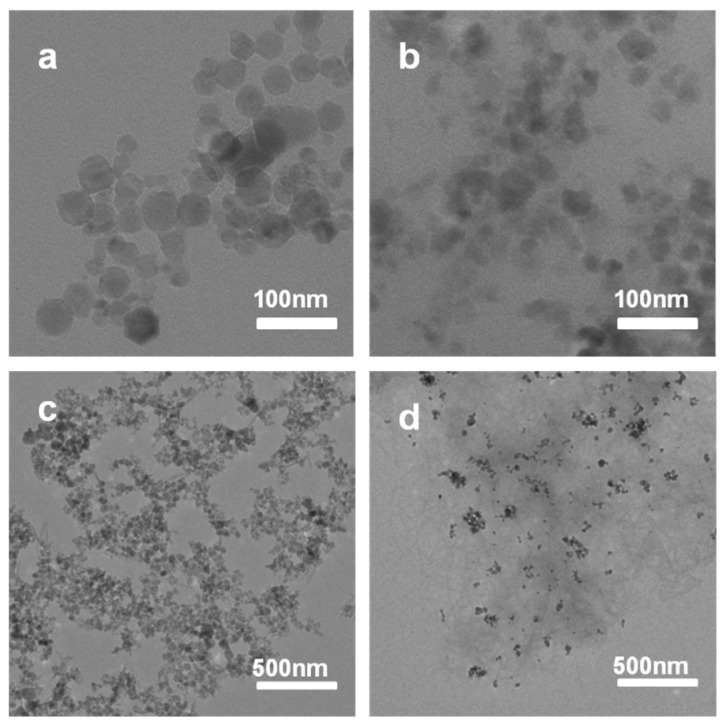
TEM micrographs of (**a**) and (**c**) the CA-Fe_3_O_4_ NPsdispersed in water at different magnification; (**b**) and (**d**) the CA-Fe_3_O_4_@Zn/AMP gels dissolved in water at different magnification.

Fourier transform infrared spectroscopy (FTIR) analysis was performed to characterize the interaction mechanism of magnetic NPs and nanofibers (Figure 2). The FTIR spectrum of bare CA-Fe_3_O_4_ NPs showed a strong band at 577.3 cm^−1^ (Figure 2, curve c), corresponding to the characteristic vibration of the Fe-O [46]. The absorption bands of PO^3−^ of CA-Fe_3_O_4_@Zn/AMP nanofibers (Figure 2, curve a) at 1070.0 cm^−1^ and 980.0 cm^−1^ shifted to higher wavenumbers, compared with the mixture of CA-Fe_3_O_4_ NPs and AMP, which indicated that the phosphate and the adenine base of AMP were involved since the interaction between nanofibers and magnetic NPs [43]. Besides, the FTIR spectra of CRL and CA-Fe_3_O_4_@Zn/AMP also demonstrated that free CRL had successfully coordinated with the Zn/AMP nanofiber (Figure S1). Then, X-ray diffraction (XRD) patterns were used to analyze the crystalline structure of CA-Fe_3_O_4_ NPs [28] (Figure S2). According to the results, the typical diffraction peaks of Fe_3_O_4_ NPs showed no change after entrapment into the Zn/AMP gels. The morphology of CA-Fe_3_O_4_ NPs in Zn/AMP nanofibers was analyzed by transmission electron microscope (TEM) analysis [17,28]. In Figure 3a,c, the prepared magnetic NPs appeared as pentagons and hexagons, and their good dispersity was evident. The TEM of CA-Fe_3_O_4_ NPs appeared fuzzy after embedding into the Zn/AMP gels, which further proved that nanofiber-like CPs grew around the magnetic NPs (Figure 3b). Besides, as shown in Figure 3d, CA-Fe_3_O_4_ NPs could disperse well in nanofibers.

Since CA-Fe_3_O_4_ NPs were successfully entrapped into the Zn/AMP hydrogel nanofiber, we next studied the co-encapsulation property of CA-Fe_3_O_4_@Zn/AMP gels on enzymes. Here, we employed CRL as the guest protein. The co-immobilization process was performed by mixing AMP, CA-Fe_3_O_4_ NPs and free CRL solution in HEPES buffer (10 mM, pH 7.4). At last, ZnCl_2_ (50 mM) was added quickly [47]. Immobilized enzymes were collected by a magnet and the encapsulation rate was calculated according to the Bradford method. The amount of CA-Fe_3_O_4_ NPs would not have an effect on the encapsulation ratio of guest protein in nanofiber gels (Figure S3), while the immobilized CRL recovery activity increased with the increasing of CA-Fe_3_O_4_ NPs amounts (Figure S4).

Next, the catalytic activity of the immobilized CRL was verified on the hydrolysis of *p*-NPP. Cytidine 5′-monophosphate (CMP) and guanosine 5′-monophosphate (GMP) were also used as coordinating molecules to prepare a particle-structured co-immobilized matrix. Note that in all assays reported here, the relative activity was defined as the activity ratio of encapsulated CRL and equivalent free CRL. Compared with free enzymes, the activity of CRL and CA-Fe_3_O_4_@Zn/AMP nanofibers increased about 10% (Figure S5).

**Figure 4:**
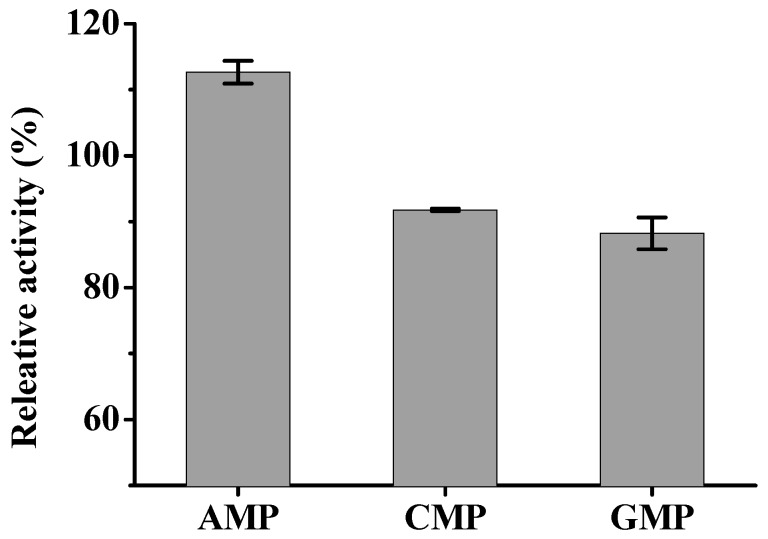
Relative activity of immobilized CRL by Zn^2+^ with different nucleotide (AMP, CMP and GMP).

However, immobilized enzymes showed a poor catalytic activity with GMP and CMP as ligands (Figure 4). This might be due to the solid state of Zn/GMP and Zn/CMP nanoparticles [42], and major enzyme molecules would be entrapped inside the composites. Only Zn/AMP nanofibers showed hydrogel-liked nanofiber structure. Thus, the porous nano-tunnel structure of Zn/AMP could offer a good substrate accessibility for the enzyme to function [43].

**Figure 5:**
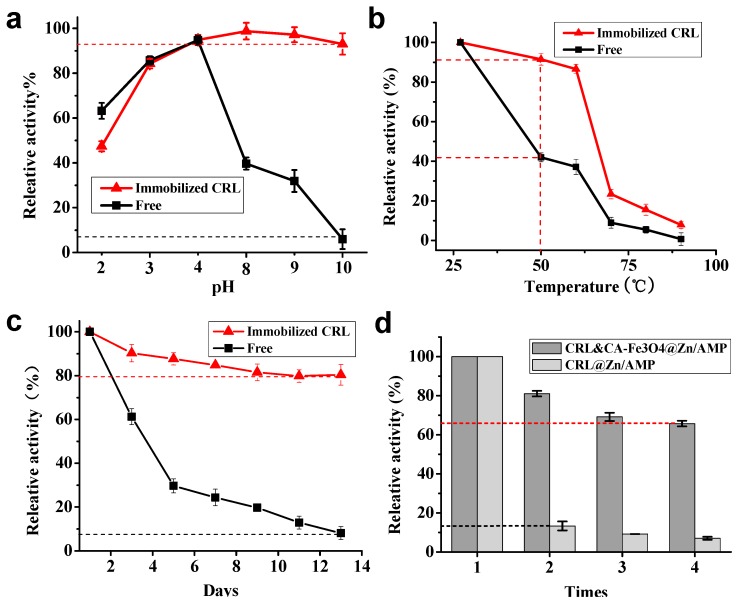
Comparison of pH (**a**), thermal (**b**), and long-term (**c**) stability of CRL&CA-Fe_3_O_4_@Zn/AMP nanofibers with equivalent free enzymes; (**d**) the recycling performance of immobilized enzymes with or without Fe_3_O_4_ NPs.

Enzymic stabilization is the most important property in enzyme commercial applications. Thus, we compared the pH, thermal and long-term storage stability of immobilized CRL with equal amount of free enzyme. The pH stability was determined out by exposing enzyme solutions to different extreme pH solutions for 6 h, respectively. The results (Figure 5a) showed that the immobilized CRL could maintain around 100% residual activity in alkaline solution, while the relative activity of free CRL decreased quickly, especially at pH 10.0.

This might be due to the CA-Fe_3_O_4_@Zn/AMP nanofibers enhancing the enzyme rigidity and improving the CRL tolerance to high pH conditions [48]. Thus, the CA-Fe_3_O_4_@Zn/AMP nanofibers could protect CRL from deactivation in alkaline solution [43]. The thermal stability (Figure 5b) of enzyme solution was evaluated in a 50–90 °C water bath [49]. Although the activity of CRL and CA-Fe_3_O_4_@Zn/AMP nanofibers and free CRL both decreased as the temperature was increased from 25 °C to 90 °C, the immobilized CRL still retained higher relative activity than that of free CRL at 50 °C, indicating that the support could improve the heat resistance of CRL solution at high temperature. The possible explanation is the CA-Fe_3_O_4_@Zn^2+^/AMP grows around each protein via multiple forces, such as hydrogen bonding, aromatic stacking, van der Waals force [42] and electrostatic attraction [50], which protects the CRL from unfolding and conformation transitions at high temperature [51,52]. The storage stability study was performed in a 4 °C freezer for 13 days and the activity of enzyme was measured every day to evaluate the effect of immobilization on the enzyme stability. At the end of observation period, the relative activity of CRL and CA-Fe_3_O_4_@Zn/AMP nanofibers was 80% after they were stored 13 days (Figure 5c), while free CRL became almost inactive, with a relative activity of 8%. Based on these results, we deduced that the stability of immobilized CRL might be ascribed to the encapsulation of enzymes in CA-Fe_3_O_4_@Zn/AMP, which could reduce the deactivation of CRL.

In order to estimate the reusability of immobilized CRL, the immobilized enzyme was recycled and reacted with fresh substrate for the next reaction [27]. Results indicated that CRL and CA-Fe_3_O_4_@Zn/AMP nanofibers could reach a relative activity of more than 60% after being reused four times, while only 14.16% for CRL@Zn/AMP nanofibers was observed after reuse the second time [53] (Figure 5d). The higher reusability of CRL and CA-Fe_3_O_4_@Zn/AMP nanofibers might due to the mild magnetic recovery process, as the traditional centrifugal separation method is so severe that the structure of Zn/AMP hydrogel nanofiber was damaged. On the other hand, the properties of CA-Fe_3_O_4_@Zn/AMP hydrogels also contributed to the reusability improvement, protecting the structure of CRL and improving the stability of the immobilized CRL against pH, temperature and storage time.

## 3. Materials and Methods

### 3.1. General Remarks

Transmission electron microscopy (TEM) analysis was performed on a H-800 transmission electron microscope (Hitachi, Tokyo, Japan) by pipetting a drop of the aqueous solution of samples onto a 230 mesh holy carbon copper grid. The crystal structure of all samples were determined by powder X-ray diffraction (D8 Advance X-Ray diffractometer, Bruker, Karlsruhe, Germany) with a Cu Kα anode (λ = 0.15406 nm) at 40 kV and 40 mA. FTIR spectra were recorded on a FTIR spectrometer (8700/Continuum XL Imaging Microscope, Nicolet, Waltham, MA, USA). The spectra were collected between 400 and 4000 cm^−1^.

### 3.2. Materials

Lipase from *Candida rugosa (Type VII)* was purchased from Sigma-Aldrich (St. Louis, MO, USA). Adenosine 5′-monophosphate (AMP) disodium salt, cytidine 5′-monophosphate (CMP) disodium salt, guanosine 5′-monophosphate (GMP) disodium salt, FeCl_3_·6H_2_O, FeCl_2_·4H_2_O, *N*-2-hydroxyethylpiperazine-*N*′-2-ethyl sulfonic acid (HEPES) and *p*-nitrophenylpalmitate (*p*-NPP) were purchased from Aladdin (Shanghai, China). Zinc chloride and NH_3_·H_2_O were from Beijing Chemical Works (Beijing, China). Bovine serum albumin (BSA) was from Sinopharm Chemical Reagent Co., Ltd. (Shanghai, China). Milli-Q water was used to prepare all the buffers and solutions. All other reagents and solvents were of analytical grade.

### 3.3. Preparation of Nanoparticles

#### 3.3.1. Preparation of Fe_3_O_4_ Nanoparticles

The synthesis of Fe_3_O_4_ nanoparticles was carried by a reported method [54]. FeCl_3_·6H_2_O (3.25 g) and FeCl_2_·4H_2_O (1.195 g) were dissolved with Milli-Q water (50 mL), then the aqueous solution was heated up and kept at 50 °C for 30 min with vigorous stirring under a N_2_ atmosphere. After that, NH_3_·H_2_O (6.25 mL) was added to the reaction system and the temperature was raised to 75 °C with continuous stirring for another 60 min. The products were collected by a magnet and washed with Milli-Q water and ethanol three times. Finally, the collected solid products were lyophilized in a freeze dryer.

#### 3.3.2. Preparation of Citric Acid Encapsulated Fe_3_O_4_ Nanoparticles

The citric acid encapsulated Fe_3_O_4_ nanoparticles were prepared according to a reported method [45]. Briefly, Fe_3_O_4_ nanoparticles (1.0 g) were mixed with Milli-Q water (50 mL) in a 100 mL round-bottom flask with stirring under ultrasonication for 15 min. Then the solution was heated to 90 °C and 2.0 M citric acid (4.5 mL) was added into the flask. After 90 min, the reaction was terminated. The whole experimental process was carried out under nitrogen. The collecting and washing steps were the same as the above ones.

#### 3.3.3. Preparation of Magnetic CPs

Since the modification of Fe_3_O_4_ NPs with citric acid could improve the stability and dispersibility of Fe_3_O_4_, bare Fe_3_O_4_ nanoparticles were replaced by CA-Fe_3_O_4_ NPs in the studies. All chemicals including zinc chloride, AMP and CA-Fe_3_O_4_ NPs were diluted with Milli-Q water. The magnetic hydrogel was obtained by mixing AMP (25 mM, 200 µL) with HEPES buffer (10 mM, pH 7.4), CA-Fe_3_O_4_ NPs solution (5 mg/mL, 50 µL), HEPES buffer (10 mM, pH 7.4, 650 µL) and ZnCl_2_ solution (100 µL, 50 mM) quickly and vortexing the mixture completely. All the samples were separated by the external magnet.

### 3.4. Enzyme Immobilization

The enzyme immobilization procedures were performed as follows: 25 mM AMP (200 µL) was mixed with HEPES buffer (10 mM, pH 7.4) and CA-Fe_3_O_4_ NPs solution (5 mg/mL, 50 µL). Then, CRL solution (1 mg/mL, 50 µL) and ZnCl_2_ solution (50 mM, 100 µL) were added, with quick mixing by vortexing. After 1 h, the immobilized CRL was collected by a magnet.

### 3.5. Protein Concentration Assay

The protein concentration assay was carried out according to the Bradford method [55]. Samples were mixed with Bradford reagent, and the absorbance was measured at 595 nm. A mixture of Bradford reagent and ultrapure water was used as the control. Bovine serum albumin (BSA) was used as a standard for the calibration curve to estimate the protein content [56].

### 3.6. Activity Assay of Enzymes

Lipase activity was determined by the hydrolysis of *p*-NPP. One mL of *p*-NPP solution (500 µg/mL) was mixed with phosphate buffer solution (2 mL, 50 mM, pH 7.4), then added free CRL (100 μL, 100 μg/mL) or immobilized CRL suspension (100 μL, containing 100 μg/mL CRL) were added into the reaction system. The mixtures were incubated at 37 °C water bath for 5 min. Then the reaction was terminated by adding 2 mL of Na_2_CO_3_. The reaction was monitored with a UV/Vis spectrometer at 410 nm.

### 3.7. Enzyme Stability Test

Stability experiments of CRL and CA-Fe_3_O_4_@Zn/AMP gels including thermal stability, pH stability, storage and recycling stability were performed [27]. For pH stability, the suspension of immobilized CRL nanofibers and free enzymes in HEPES buffer (pH 7.4, 10 mM) were placed into six different extreme pH solutions (pH 2–4, 8–10) for 6 h, respectively. To test thermal stability, the immobilized enzyme nanofibers and free enzymes solution were evaluated in a 50–90 °C water bath for 30 min [49]. The storage stability of the immobilized and free CRL in HEPES buffer (10 mM, pH 7.4) at 4 °C were carried out by measuring the residual enzymatic activity after 13 days of storage [17,48]. For estimating the reusability, the immobilized enzyme was collected from the reaction media by a magnet and reused on fresh substrate for the next reaction [27]. During all stability experiments, the residual enzymatic activity was measured by the method described above and the initial activity of all enzymes was regarded as 100%.

## 4. Conclusions

We have reported a convenient method for preparing magnetic response immobilized enzyme by co-entrapping CA-Fe_3_O_4_ nanoparticles and CRL simultaneously with Zn/AMP nanofiber supports. The CA-Fe_3_O_4_@Zn/AMP gels could retain the original fibrillar structure of Zn/AMP gels. Magnetic nanomaterials provided convenience for enzymatic immobilization and separation. Moreover, CA-Fe_3_O_4_@Zn/AMP gels showed excellent pH stability, thermal stability and long-term stability.

## Supplementary Materials

Supplementary materials can be accessed at: http://www.mdpi.com/1420-3049/22/1/179/s1.
