# Supplementary material for: Co-Immobilization of Enzymes and Magnetic Nanoparticles by Metal-Nucleotide Hydrogelnanofibers for Improving Stability and Recycling
Source: Molecules. 2017 Jan 23;22(1):179. doi: 10.3390/molecules22010179 (PMC6155653; doi:10.3390/molecules22010179)
Supplement: Supplementary file 1 [file molecules-22-00179-s001.pdf]

Supplementary Materials: Co-Immobilization of Enzymes and Magnetic Nanoparticles by Metal-Nucleotide Hydrogel Nanofibers for Improving Stability and Recycling

Chunfang Li, Shuhui Jiang, Xinying Zhao and Hao Liang

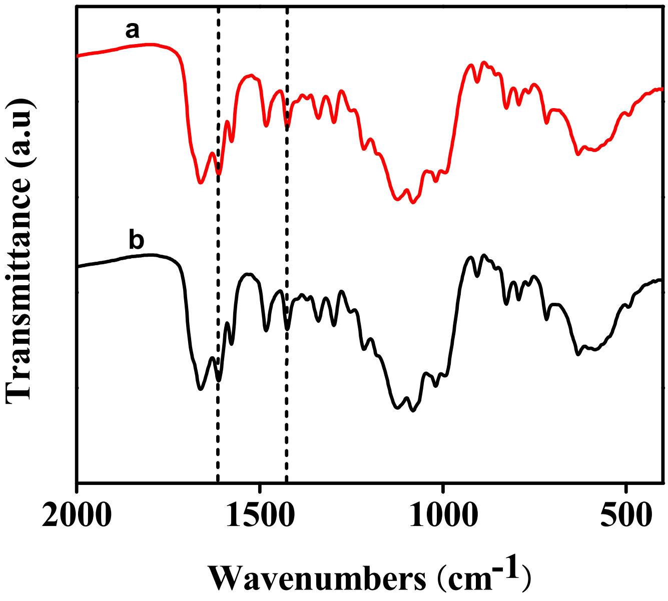

Figure S1. The FTIR spectra of CRL&CA-Fe_3O_4@Zn/AMP gels (curve **a**), and CA-Fe_3O_4@ Zn/AMP gels (curve **b**).

Compared with curve (b) in Figure S1, the absorption bands at 1425.2 cm^{-1} and 1662 cm^{-1} in curve (a) occurred stretching vibrations, attributing to the N-H bending vibrations of amide II and amide I of the protein [1], indicating that CRL was encapsulated into CA-Fe_3O_4@Zn/AMP gels successfully [1,2].

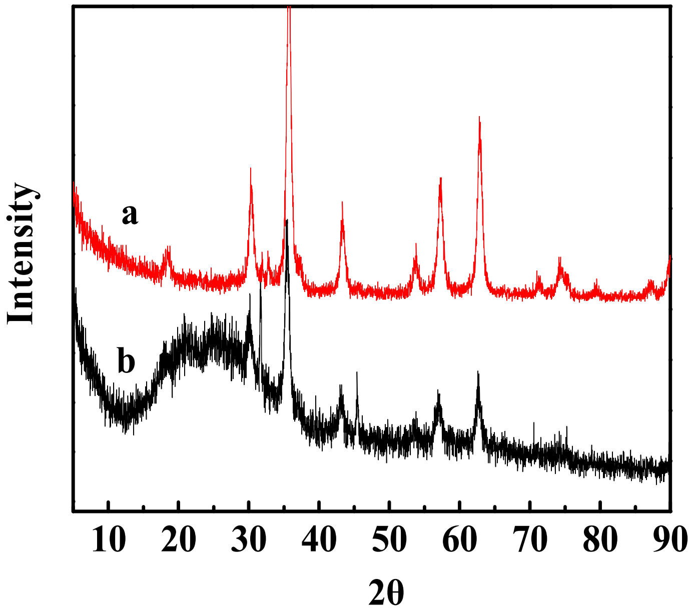

Figure S2. XRD spectra of (**a**) CA-Fe_3O_4 NPs and (**b**) CA-Fe_3O_4@Zn/AMP nanofibers.

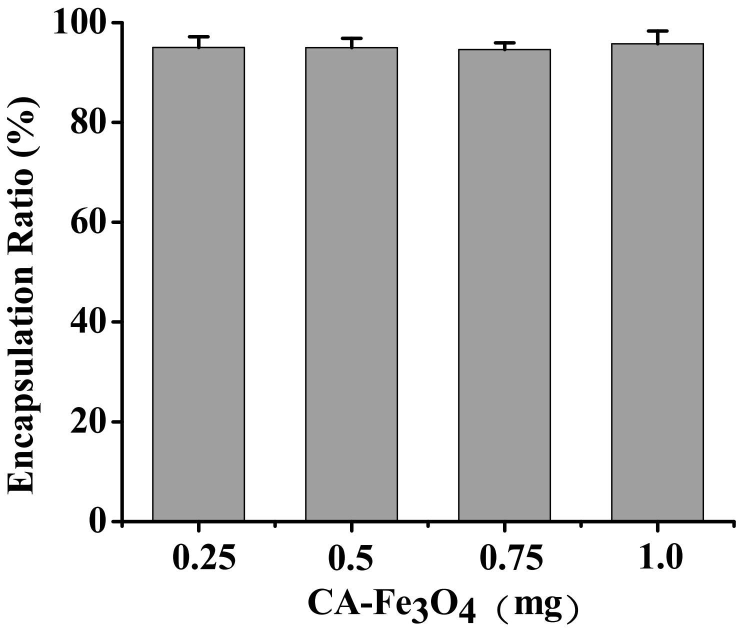

Figure S3. Encapsulation ratio of CRL@Zn/AMP at different amounts of CA-Fe_3O_4 NPs.

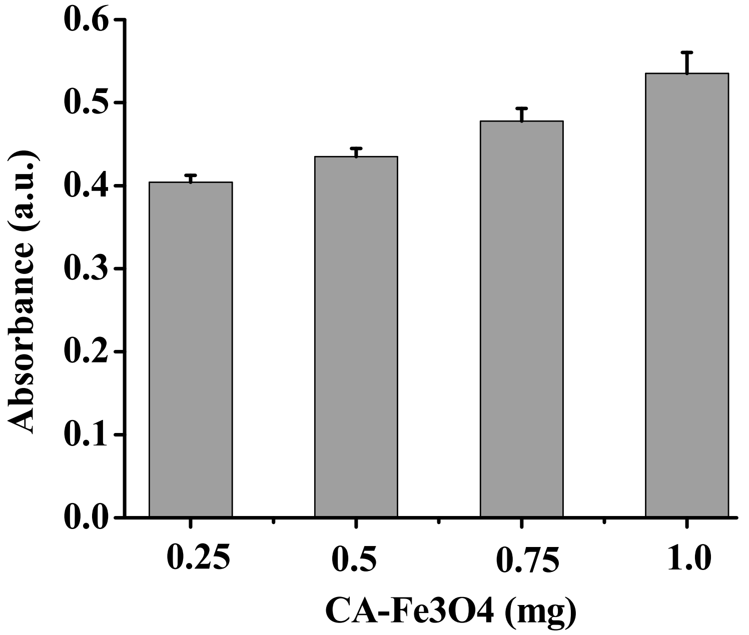

Figure S4. The catalytic activity of CRL&CA-Fe_3O_4@Zn/AMP nanofibers at different amounts of CA-Fe_3O_4 addition in the immobilization process.

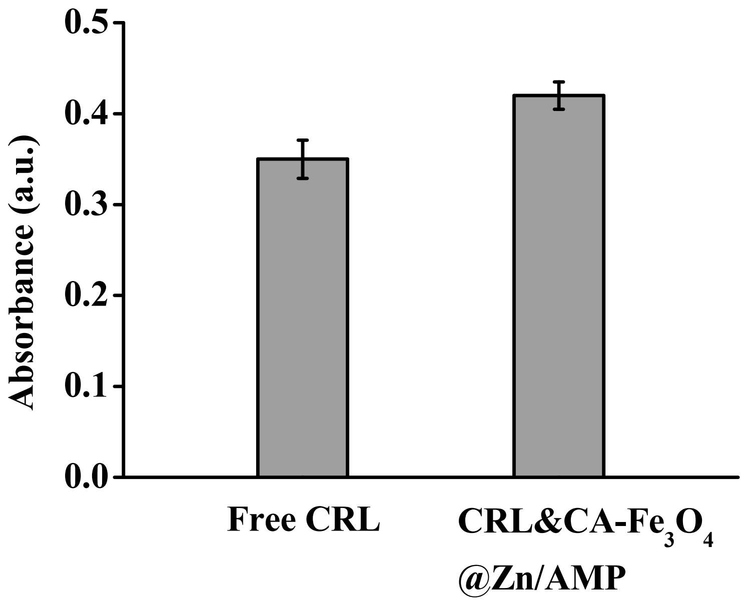

Figure S5. The catalytic activity of free CRL and CRL&CA-Fe_3O_4@Zn/AMP nanofibers.

References

1. Liang, H.; Jiang, S.; Yuan, Q.; Li, G.; Wang, F.; Zhang, Z.; Liu, J. Co-immobilization of multiple enzymes by metal coordinated nucleotide hydrogel nanofibers: improved stability and an enzyme cascade for glucose detection. *Nanoscale* **2016**, *8*, 6071–6078.
2. Ghorbani-Choghamarani, A.; Tahmasbi, B.; Moradi, P. Palladium-*S*-propyl-2-aminobenzothioate immobilized on Fe_3O_4 magnetic nanoparticles as catalyst for Suzuki and Heck reactions in water or poly (ethylene glycol). *Appl. Organomet. Chem.* **2016**, *30*, 422–430.
